# The Protection of Selenium against Cadmium-Induced Cytotoxicity via the Heat Shock Protein Pathway in Chicken Splenic Lymphocytes

**DOI:** 10.3390/molecules171214565

**Published:** 2012-12-07

**Authors:** Xi Chen, Yi-Hao Zhu, Xin-Yue Cheng, Zi-Wei Zhang, Shi-Wen Xu

**Affiliations:** Department of Veterinary Medicine, Northeast Agricultural University, Harbin 150030, China

**Keywords:** cadmium, selenium, heat shock protein, chicken splenic lymphocytes, protective effects

## Abstract

Cadmium (Cd) is a heavy metal that poses a hazard to animal health due to its toxicity. Selenium (Se) is an important nutritional trace element. However, the potential protective effects of Se against Cd-induced toxicity remain to be elucidated. To investigate the cytotoxicity of Cd on bird immunocytes *in vitro* and the protective effects of Se against exposure to Cd, chicken splenic lymphocytes received Cd (10^−6^ mol/L), Se (10^−7^ mol/L), and the mixture of 10^−7^ mol/L Se and 10^−6^ mol/L Cd and were incubated for 12 h, 24 h, 36 h, 48 h, respectively. The transcription of heat shock protein (HSP) 27, HSP40, HSP60, HSP70 and HSP90 mRNA was tested by fluorescence quantitative PCR. The results showed that the mRNA expression of HSPs exposed to 10^−6^ mol/L Cd showed a sustained decrease at 12–48 h exposure. A statistically significant increase in the mRNA expression of HSPs in the case of Se group was observed, as compared to the control group of chicken splenic lymphocytes. Concomitantly, treatment of chicken splenic lymphocytes with Se in combination with Cd enhanced the mRNA expression of HSPs which were reduced by Cd treatment. This indicated that the protective effect of Se against the toxicity of Cd might, at least partially, be attributed to stimulation of the level of HSPs.

## 1. Introduction

Cadmium (Cd) is a heavy metal known to be both ubiquitous in natural environments and extremely toxic. Cd pollution has been identified as a potential health threat to wildlife. Its technological utilization has led to increasing levels in the environment and also in the human body [1]. From a global perspective Cd pollution has been increasing as human activities have enormously aggravated Cd pollution in diverse ecosystems. Present studies indicate that Cd is highly toxic towards multiple organs and a potent immunotoxicant. The immunosuppressive properties of Cd were demonstrated both *in vivo* and *in vitro* on humans and rodents [2,3,4]. Numerous studies have indicated that Cd exposure reduced the cell viability of lymphocytes in rodents and humans, and also induced oxidative stress, apoptotic and necrotic death [5,6]. The levels of Cd in the environment vary widely due to its ability to be transported through air, water, and soil. Animals normally absorb Cd into the organism either by ingestion or inhalation. The organism does not have an effective Cd elimination pathway and as a result the biological half-life of Cd in the organism is estimated to be 15–20 years. Cd causes pleiotropic effects on organisms at both the molecular and cellular levels. It binds to cysteine residues of proteins and induces oxidative stress [7].

Considering the high sensitivity of the immunologic function to Cd insult, prevention and/or therapeutic interventions are of major concern [8]. Several elements have been shown to have a protective effect against Cd-induced injury, among which selenium (Se) is considered one of the most efficient. Se is an important nutritional trace element [9], which contributes significantly to host immune responses and antioxidant protection [10]. Se functions in the body as an antioxidant, in thyroid hormone metabolism, redox reactions, reproduction, and immune function [11]. Se plays a role in protecting cells against free radicals and oxidative stress [12]. Numerous studies have shown that Se can protect against Cd toxicity in mammals *in vitro* and *in vivo* [13,14], but the effects of Se on Cd-induced cell injury in chicken splenic lymphocytes have not been studied.

Stressors, e.g., anoxia, and heavy metal ions, rapidly induce the synthesis of heat shock proteins (HSPs), which are also expressed constitutively in poultry species [15]. Many HSPs, especially the 70 kDa family (HSP70), are expressed in response to diverse stressors, and increased synthesis of these inducible proteins is involved in the protection of stressed cells and organisms [16]. Numerous studies have documented an increase in levels of individual members of the HSP family following exposure of cells to toxic chemicals [17,18,19], but there are few studies in which multiple HSPs have been investigated. Exposure of cells to a chemical stress elicits an up-regulation of a number of cytoprotective systems [20], amongst which the heat shock response is one of the most studied [21]. In this response, the synthesis of HSPs is up-regulated; these proteins play a role in maintaining protein structure/function by acting as molecular chaperones to assist the correct folding of damaged or newly synthesized proteins [22]. As stress response proteins, HSP act as a ‘danger signal’ to immune cells, promoting immune responses that are involved in protection of the cytoplasm components [23]. It is widely accepted that HSPs are essential factors for activating many signal proteins such as steroid hormone receptors, cell cycle kinase Cdk4 and important immune defense reactions [24].

A recent study indicated that Cd could be accumulated in the organism, by which higher concentrations of materials accumulate in organisms higher up in the food chain [25]. Up-regulation of the expression of HSPs by Cd in various tissues is well documented [26]. This increased expression of HSPs is classically known to protect cells against a variety of stimulants that can damage or denature cellular proteins. The present study was, therefore, carried out to determine whether Se supplementation could have a protective effect against the Cd-induced toxic effect in chicken splenic lymphocytes through the HSP pathway.

## 2. Results and Discussion

**Figure 1:**
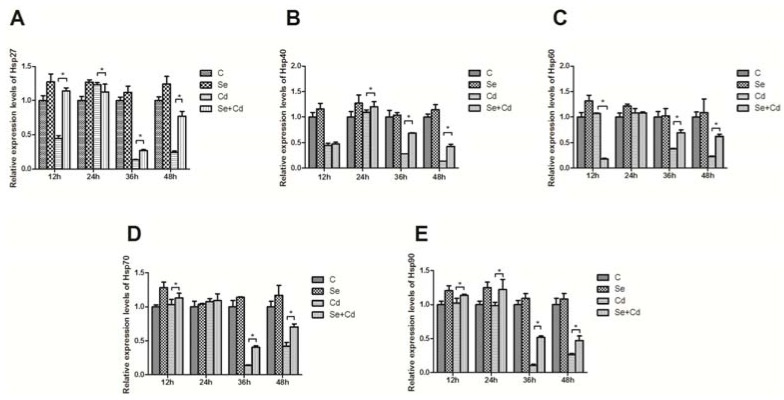
Effects of Se on Cd-induced changes in the mRNA levels of HSP. Panels **A**–**E** represent the effects of Se on Cd-induced changes in the mRNA levels of HSP27, HSP40, HSP60, HSP70, HSP90, respectively. The relative mRNA levels from the C groups were used as the reference values, the * with connecting lines indicated that there were significant differences (*p* < 0.05) between any two groups. Each value represented the mean ± SD of 5 individuals.

Cd stimulation can cause a series of immune responses in organisms to maintain cells in a physiological balance, normal signal conduction and immune related gene expression, which can improve an organisms’ ability to resist damage. Hanson [27] suggested that even very low levels of Cd exposure during gestation could result in long term detrimental effects on the immune system. Cd has previously been shown to increase HSP levels in various cell-types, and appears to mediate toxicity through a common oxidative stress mechanism [28]. Gottschalg’s research showed that exposure of hepatocytes to CdCl_2_ elicited significant up-regulation of HSPs expression [29]. *In vivo* and *in vitro* studies in mice have shown that splenocytes are more sensitive to Cd exposure than are thymocytes [3,5]. However, no literature is available regarding the toxic effects of Cd through HSP pathway on the splenocytes of birds. Thus the toxicity of Cd to chicken splenic lymphocytes was estimated by determination of HSP response. In the present study, the mRNA level of HSP27, HSP40, HSP60, HSP70 and HSP90 exposed to 10^−6^ mol/L Cd showed a sustained decrease at 12–48 h exposure (Figure 1A-E), which was not in accordance with previous data indicating that Cd exposure could increase the expression of HSP in three hepatic-derived cell culture systems (rat hepatoma FGC4 cell line, rat hepatocytes, human hepatoma HepG2 cell line) [29]. Clearly, the mRNA expression of HSPs was inhibited by Cd during the experimental stage. One possible explanation is that damage in cells can limit HSP synthesis and result in cellular energy deficiency. Such a decrease in the metabolic capacity may be due to Cd toxicity.

Oxidative stress seems to be crucial in the etiology of Cd-induced immunotoxicity in humans and animals. Thus, antioxidant therapy is considered an important approach to intervention of Cd toxicity. Se is among the important nonenzymatic antioxidant defense systems, which may modulate its toxicity by an antioxidative mechanism. Se acts as a component of the unusual amino acids selenocysteine (Se-Cys) and selenomethionine (Se-Met), and functions as a co-factor for the reduction of antioxidant enzymes, including glutathione peroxidases and certain forms of thioredoxin reductase. Se deficiency is associated with increased levels of oxidative stress and its related diseases [30,31]. Various types of reactive oxygen species (ROS) are formed *in vivo*, primarily as a result of aerobic metabolism. Many of these are powerful oxidising agents, capable of damaging DNA and other biomolecules. Se has been attributed the ability to scavenge ROS, while the glutathione peroxidase family of enzymes (GSH-PX) are capable of catalysing reactions to remove ROS. Se was also found to protect cells through HSP pathway [32]. In the present study, a statistically significant increase in the mRNA expression of HSPs in the case of Se group was observed, as compared to the control group of chicken splenic lymphocytes, which was in accordance with previous data. It indicated that Se might provide protection of cells through the HSP pathway. A study carried out on animals has revealed that Se exerted a protective effect against the toxicity of cadmium [13]. The combination of vitamin C, vitamin E, and Se showed a protective effects against cadmium toxicity in rat [33]. In Zhou’s study, pretreatment with different concentrations of Na_2_SeO_3_ blocked CdCl_2_-induced ROS generation and suppressed CdCl_2_-induced apoptosis of LLC-PK_1_ cells [34]. Our previous research suggested that the toxic effect of cadmium on the testes was ameliorated by Se [35]. In addition, to the best of our knowledge, no comprehensive study concerning the protective effect of Se on Cd-induced change of HSP genes has been reported. In the present study, treatment of chicken splenic lymphocytes with Se in combination with Cd enhanced the mRNA expression of HSPs which were reduced by Cd treatment (Figure 1A–E). The protective effect of Se might be due to an interaction of Se with Cd, forming biologically inactive cadmium-selenide complexes [13]. The upregulation of HSP genes observed upon Se exposure might be a protective and adaptive response by the cells.

## 3. Experimental

### 3.1. Preparation of Chicken Splenic Lymphocytes Suspension and Treatment

All procedures used in this experiment were approved by the Institutional Animal Care and Use Committee of Northeast Agricultural University. Spleens were dissected from Isa brown cocks (60 days old) and were collected aseptically and placed in sterile phosphate-buffered saline (PBS, 0.1 M phosphate buffer with 0.85% NaCl, pH 7.2). Single cell suspension was prepared by gently pushing the splenic pulp through a sterile stainless steel mesh with a pore size of 100 micron. Cells were washed and resuspended in 5 mL sterile PBS and then layered over 5 mL lymphocytes separation medium (Tian Jin Hao Yang Biological Manufacture. CO. LTD., Tianjin, China). The splenocyte preparations were enriched by centrifugation (2,000 × *g*) for 15 min at 18 °C. Cells were recovered from the interface, resuspended, and washed twice in 8 mL cell culture medium (RPMI 1640, Gibco, Grand Island, NY, USA). The cells were suspended in complete cell culture medium [RPMI 1640 containing HEPES and 2 mM glutamine, supplemented with 10% fetal bovine serum (FBS, Gibco) and 1% antibiotic-antimycotic solution (Sigma, Santa Clara, CA, USA)]. Splenic lymphocytes density was adjusted to 1.5 × 10^6^ cells/mL and the viability of the freshly isolated cells was always above 95% (trypan blue exclusion test). For the monitoring of various parameters in the present investigation, the control group was incubated for 12 h, 24 h, 36 h, 48 h without reagents. 10^−6^ mol/L Cd (Cd group), 10^−7^ mol/L Se (Se group), and the mixture of 10^−7^ mol/L Se and 10^−6^ mol/L Cd (Se+Cd group) were added while cells at logarithmic growth phase and incubated for 12 h, 24 h, 36 h, 48 h, respectively. The concentrations of Cd and Se used in this study were according to our previous studies [36,37].

### 3.2. Quantification of HSPs mRNA

Total RNA was isolated from cells using Trizol reagent according to the manufacturer’s instructions (Invitrogen, Grand Island, NY, USA). The RNA concentrations were determined using the GeneQuant 1300. Reverse transcription reaction (40 µL) consisted of the following: 10 µg of total RNA, 1 µL of M-MLV reverse transcription, 1 µL of RNase inhibitor, 4 µL of dNTP, 2 µL of Oligo dT, 4 µL of dithiothreitol, and 8 µL of 5×buffer. The procedure of the reverse transcription was according to the manufacturer’s instructions (Invitrogen). The reverse transcription products (cDNA) were then stored at −20 °C for PCR.

To design primers, we used the chicken HSP27, HSP40, HSP60, HSP70 and HSP90 mRNA GenBank sequence with accession number of NM_205290, NM_001199325, NM_001012916, NM_001006685, NM_001109785, respectively. Chicken β-actin (GenBank accession number L08165) as a housekeeping gene was used as an internal reference. Primers (Table 1) were designed using the Oligo 6.0 Software, and were synthesized by Invitrogen Biotechnology Co. Ltd. (Shanghai, China).

**Table 1:** Gene-special primers for HSP27, HSP40, HSP60, HSP70, HSP90 and β-actin used in the real-time quantitative reverse transcription PCR.

Gene	Accession number	Primer (5′→3′)	Product size (bp)
HSP27	NM_205290	5'-ACACGAGGAGAAACAGGATGAG-3'	158 bp
		5'-ACTGGATGGCTGGCTTGG-3'	
HSP40	NM_001199325	5'-GGGCATTCAACAGCATAGA-3'	151 bp
		5'-TTCACATCCCCAAGTTTAGG-3'	
HSP60	NM_001012916	5'-AGCCAAAGGGCAGAAATG-3'	208 bp
		5'-TACAGCAACAACCTGAAGA-3'	
HSP70	NM_001006685	5'-CGGGCAAGTTTGACCTAA-3	250 bp
		5'-TTGGCTCCCACCCTATCTCT-3'	
HSP90	NM_001109785	5'-TCCTGTCCTGGCTTTAGTTT-3'	143 bp
		5'-AGGTGGCATCTCCTCGGT-3'	
β-actin	L08165	5'-CCGCTCTATGAAGGCTACGC-3'	128 bp
		5'-CTCTCGGCTGTGGTGGTGAA-3'	

Real-time quantitative reverse transcription PCR was used to detect the expression of HSP27, HSP40, HSP60, HSP70 and HSP90 gene in cells by using SYBR Premix Ex TaqTM (Takara, Dalian, China), and real time PCR work was done in the ABI PRISM 7500 real-time PCR system (Applied Biosystems, Carlsbad, CA, USA). The program was 1 cycle at 95 °C for 30 s and 40 cycles at 95 °C for 5 s and at 60 °C for 34 s. Dissociation curves were analyzed by Dissociation Curve 1.0 Software (Applied Biosystems) for each PCR reaction to detect and eliminate the possible primer-dimer and non-specific amplification. The mRNA relative abundance was calculated according to the method of Pfaffl [38]. Results were expressed as ΔΔCt in which the normalization formula is target amount = 10^ΔCT,T/AT-ΔCT,R/AR^, where ΔCT,T = Ct (HSP sample), ΔCT,R = CT (β-actin sample), AT = slope of standard curve (HSPs), AR = slope of standard curve (β-actin).

### 3.3. Statistical Analyses

Statistical analysis of all data was performed using SPSS for Windows (version 13; SPSS Inc., Chicago, IL, USA). When a significant value (*p* < 0.05) was obtained by one-way analysis of variance, further analysis was carried out. All data showed a normal distribution and passed equal variance testing. Differences between means were assessed using Tukey’s honestly significant difference test for post hoc multiple comparisons. Data are expressed as the mean ± standard deviation.

## 4. Conclusions

The present results indicated that Cd exposure impaired the mRNA expression of HSPs in chicken splenic lymphocytes. Moreover, the protective effect of Se against the toxicity of Cd might, at least partially, be attributed to stimulate the level of HSPs. Therefore, Se can be considered a potential therapeutic nutrient to protect against toxicity induced by Cd.
